# Development of Timd2 as a reporter gene for MRI

**DOI:** 10.1002/mrm.25750

**Published:** 2015-05-15

**Authors:** P. Stephen Patrick, Tiago B. Rodrigues, Mikko I. Kettunen, Scott K. Lyons, André A. Neves, Kevin M. Brindle

**Affiliations:** ^1^ Department of BiochemistryUniversity of CambridgeCambridgeUnited Kingdom; ^2^ Cancer Research UK Cambridge Institute, University of CambridgeCambridgeUnited Kingdom

**Keywords:** ferritin, manganese, T_1_‐weighted, T_2_‐weighted, reporter gene

## Abstract

**Purpose:**

To assess the potential of an MRI gene reporter based on the ferritin receptor Timd2 (T‐cell immunoglobulin and mucin domain containing protein 2), using T_1_‐ and T_2_‐weighted imaging.

**Methods:**

Pellets of cells that had been modified to express the Timd2 transgene, and incubated with either iron‐loaded or manganese‐loaded ferritin, were imaged using T_1_‐ and T_2_‐weighted MRI. Mice were also implanted subcutaneously with Timd2‐expressing cells and the resulting xenograft tissue imaged following intravenous injection of ferritin using T_2_‐weighted imaging.

**Results:**

Timd2‐expressing cells, but not control cells, showed a large increase in both R_2_ and R_1_ in vitro following incubation with iron‐loaded and manganese‐loaded ferritin, respectively. Expression of Timd2 had no effect on cell viability or proliferation; however, manganese‐loaded ferritin, but not iron‐loaded ferritin, was toxic to Timd2‐expressing cells. Timd2‐expressing xenografts in vivo showed much smaller changes in R_2_ following injection of iron‐loaded ferritin than the same cells incubated in vitro with iron‐loaded ferritin.

**Conclusion:**

Timd2 has demonstrated potential as an MRI reporter gene, producing large increases in R_2_ and R_1_ with ferritin and manganese‐loaded ferritin respectively in vitro, although more modest changes in R_2_ in vivo. Manganese‐loaded apoferritin was not used in vivo due to the toxicity observed in vitro. Magn Reson Med, 2015. © 2015 The Authors. Magnetic Resonance in Medicine published by Wiley Periodicals, Inc. on behalf of International Society for Magnetic Resonance in Medicine. This is an open access article under the terms of the Creative Commons Attribution License, which permits use, distribution and reproduction in any medium, provided the original work is properly cited. **Magn Reson Med 75:1697–1707, 2016. © 2015 The Authors. Magnetic Resonance in Medicine published by Wiley Periodicals, Inc. on behalf of International Society for Magnetic Resonance.**

## INTRODUCTION

A variety of MR reporter genes have been developed that are detectable in T_2_‐ and T_1_‐weighted ^1^H images 1, 2, 3, 4, 5, 6, 7, 8, 9, 10, 11, 12, 13, 14, 15, 16, 17, in ^19^F images and spectra 18, 19, 20 and in ^31^P spectra 20, 21, 22, 23, 24. Recent work has also investigated the potential of MR gene reporter systems that use hyperpolarized ^13^C‐labeled metabolites 25, 26, 27 and ^129^Xe‐based probes 28, although of these only one has been demonstrated in vivo 27. Chemical exchange saturation transfer (CEST) is another promising method for producing MR contrast for which gene reporters have been developed 29, 30, 31. The signal can be switched on and off and, because contrast agents can have exchangeable protons at different frequencies, there is the possibility of “multicolor” imaging. However, the sensitivity of detection in these systems has so far been relatively low.

Various MR reporter systems have also been devised that incorporate multimodal imaging, including LacZ 11, 14, 18, 19, 20, 32, tyrosinase 7, 13, 33, 34, 35, mbGlucBiotin 10, and BAP‐TM 9. Two of the more promising multimodal approaches use DMT1, which is a transporter for manganese 15, and Oatp1, which transports a clinically approved gadolinium‐based contrast agent 16. These produce higher levels of contrast in T_1_‐weighted images than reporters described previously, as well as functioning as radionuclide imaging reporters 16, 36 and, in the case of Oatp1, enhancing bioluminescence signal from luciferase‐expressing cells as well 37.

Ferritin, a polymeric spherical iron storage protein, has been one of the more widely used reporters 1, 2, 3, 5, 38. Accumulation of ferritin above normal background levels can be detected as a hypointense signal in T_2_‐weighted images, allowing ferritin transgene expression to be detected. However, the change in relaxivity that can be achieved by ferritin overexpression is often small due to limited iron availability and accumulation, and therefore the change in image contrast can be difficult to detect 3. One approach to improving sensitivity has been to increase iron uptake by overexpression of the transferrin receptor 4. While this increased the ability of cells to take up transferrin in vitro, the improvement in image contrast in vivo was still quite modest.

We have investigated here exploitation of another iron uptake mechanism as an MRI‐based gene reporter: the receptor Timd2 (T‐cell immunoglobulin and mucin domain containing protein 2) that mediates ferritin endocytosis 39, 40, 41. T‐cell immunoglobulin and mucin domain containing protein 2 (Timd2; also known as TIM‐2) is a murine protein that is expressed primarily on immune cells, where it plays a role in signaling. Timd2 expression elsewhere is limited, but is also found in both liver and kidney, where it functions primarily as a ferritin receptor to remove ferritin from the blood 40, as well as on oligodendrocytes, where it is upregulated during myelination to meet increased iron demands 39. Because ferritin can hold up to 4000 iron atoms in its internal cavity, compared with two atoms in transferrrin, this reporter offers a potentially more sensitive alternative to the transferrin receptor‐based reporter. In addition, the iron in ferritin can be exchanged for manganese 42, making it an effective T_1_ contrast agent 43, as well as a T_2_ contrast agent.

We demonstrate in vitro that cells expressing Timd2 and incubated with ferritin or manganese‐loaded apoferritin showed large increases in R_2_ and R_1_, respectively. Cells expressing Timd2 implanted as xenografts in mice also showed changes in R_2_ following intravenous injection of ferritin, although these changes in contrast were more modest than those observed in vitro. Due to the observed toxicity with manganese‐loaded apoferritin in vitro this agent was not pursued further in vivo. Timd2 represents another addition to the growing number of proteins that can be used as MRI‐based gene reporters.

## METHODS

### Cell Culture

All experiments were carried out using HEK 293T (human embryonic kidney) cells. Cells were cultured in 5% CO_2_, at 37°C, in Dulbecco's modified Eagle's medium (DMEM, Life Technologies), supplemented with 10% fetal bovine serum and 2 mM L‐glutamine (Life Technologies).

### Production of the Lentiviral Transfer Plasmid

A sequence encoding mStrawberry 44, E2A (amino acids QCTNYALLKLAGDVESNPGP) 45, and murine Timd2 (NCBI reference sequence NM_001161356.1), was initially assembled in a pBluescript II plasmid. The three coding sequences were arranged in frame, and the stop codon at the end of mStrawberry was mutated so that only a single stop codon at the end of Timd2 remained. The transgene was then cloned into the lentiviral transfer vector backbone plasmid pBOBI 46 (a gift from Inder Verma, Salk Institute, California). A 500 bp shortened mouse phosphoglycerate kinase (PGK) promoter was inserted upstream of this sequence. This promoter is constitutively active in mammalian cells and is homologous to the human promoter 47. The vector was sequenced to confirm that no errors had been introduced during its construction.

### Lentiviral Preparation

Replication defective vesicular stomatitis virus glycoprotein (VSV‐G) pseudotyped lentiviral vectors were produced 48, and purified 49 according to published protocols, using the assembled pBOBI‐PGK‐mStrawberry‐E2A‐Timd2 transfer plasmid and three packaging plasmids. The packaging plasmids, pMDL, pVSV‐G, and pRSV‐REV, were a gift from Inder Verma (Salk Institute, California).

### Fluorescent Labeling of Ferritin

Ten mg of horse spleen ferritin (Sigma‐Aldrich) were dissolved in sodium borate buffer (100 mM, pH 9), at a concentration of 0.044 mg/mL and reacted with fluorescein isothiocyanate (FITC, previously dissolved in dimethyl sulfoxide at 13 mg/mL) for 1 h in the dark, at room temperature, while stirring at 100 rpm. The reaction was quenched by the addition of a 500‐fold molar excess of Tris‐HCl (12 µM; pH 8). Unreacted FITC was removed by extensive buffer‐exchange into HBS buffer (HEPES‐buffered saline: 20 mM HEPES, 150 mM NaCl, pH 7.4) using 30 kDa cut‐off spin filters (GE Healthcare). FITC‐labeled ferritin was analyzed by sodium dodecyl sulfate‐polyacrylamide gel electrophoresis (SDS‐PAGE), with brightfield and fluorescence detection to confirm successful labeling.

### Confocal Microscopy

Cells were grown on sterile glass cover slips. Before imaging the growth medium was removed and the cover slips washed in phosphate‐buffered saline (PBS) and then inverted onto a glass slide with a drop of Prolong® Gold mounting medium containing DAPI (4′,6‐diamidino‐2‐phenylindole) nuclear stain (Life Technologies), and left to dry in the dark. Slides were imaged using a Leica Tandem confocal microscope with an oil objective. Images were assembled as maximum projections from Z‐stacks of slices taken at 1‐μm intervals.

### Western Blot

Cells were lysed in 5 µL of cell lysis buffer (M‐Per, Thermo Scientific) per mg of cells, according to the manufacturer's instructions. Protein concentration was estimated using the Bradford assay. Twenty µg of whole cell lysate were mixed with 4× loading buffer (NuPAGE LDS sample buffer, Life Technologies,) and 10× reduction buffer (NuPAGE sample reducing agent, Life Technologies), made up to 20 µL with PBS, and heated to 70°C for 10 min. Samples were run on a 4 to 12% PAGE gel (NuPAGE Novex Bis‐Tris gel 1 mm 10 well, Life Technologies), with a protein standard ladder (Kaleidoscope Precision Plus Protein Standards, Bio‐Rad), at 150 V for 75 min. Proteins were then blotted onto a nitrocellulose membrane at 30 V for 60 min using TGS RunBlue transfer buffer. For α‐tubulin staining (loading control), monoclonal mouse anti‐α‐tubulin (T1568, Sigma‐Aldrich) was used at 1 in 1000 dilution with overnight incubation at 4°C. For ferritin staining, polyclonal rabbit anti‐ferritin light‐chain antibody (ab69090, Abcam) was used at 1 in 500 dilution with overnight incubation at 4°C. For secondary antibody staining, horseradish peroxidase conjugated anti‐rabbit and anti‐mouse antibodies were used at 1 in 10,000 dilution with an incubation time of 45 min at room temperature. All antibodies were diluted in Tris‐buffered saline (TBS), pH 7.6, + 0.1% tween and 5% milk powder. Membranes were washed 5 times before and after all antibody incubations in TBS + 0.1% tween. For imaging, membranes were incubated in SuperSignal Chemiluminescent substrate for 5 min, then exposed to CL‐XPosure film (both from Thermo‐Scientific).

### Preparation of Manganese‐Loaded Apoferritin

Manganese‐loaded apoferritin was prepared according to 42. Briefly, horse spleen apoferritin was dissolved at a concentration of 3 µM in 5 mL AMPSO (N‐(1,1‐Dimethyl‐2‐hydroxyethyl)‐3‐amino‐2‐hydroxypropanesulfonic acid) buffer (0.05 M, pH 8.9). To this was added MnCl_2_ to a concentration of 9 mM (∼3000 manganese ions per apoferritin). The solution was left overnight, so that any large insoluble MnOOH particles not formed in the ferritin cavity would sediment, and was then buffer exchanged into HEPES‐buffered saline (pH 7.4) using a Vivaspin centrifugation filter (GE Healthcare) with molecular cut‐off weight of 30 kDa. Protein concentration was estimated using the Bradford Assay.

### Flow Cytometry

A polyclonal population of cells transduced with the mStrawberry‐Timd2 lentivirus, and untransduced cells, were incubated with various concentrations of FITC‐labeled ferritin at 37°C, washed with PBS, trypsinized, and suspended in PBS. One thousand events were recorded per population. Forward and side scatter were measured to estimate cell viability. Fluorescence at 530 nm was used to detect the presence of FITC‐ferritin. Transgenic cells were identified by increased fluorescence at 630 nm, indicating expression of mStrawberry. Flow cytometry data were acquired using a FACSCalibur flow cytometer (BD Biosciences), and analyzed using FlowJo software (Treestar).

### Preparation of Cell Pellets for MRI

A polyclonal population of HEK 293T cells transduced with the mStrawberry‐Timd2 lentivirus (multiplicity of infection of 2), or untransduced cells, were grown on 15 cm plates (Nunc), until they were ∼80% confluent. These were incubated for different lengths of time, at which their relaxivities were measured following incubation with either 75 nM horse spleen ferritin or 75 nM manganese‐loaded apoferritin. After incubation, cells were gently washed with PBS, trypsinized with 0.25% trypsin (Life Technologies), and resuspended in PBS. The cells were pelleted in Eppendorf tubes using a microcentrifuge and kept on ice before imaging.

### MRI

Experiments were carried out in a 7 Tesla (T) horizontal magnet (Oxford Instruments, UK) interfaced to a VNMRS (Varian Inc) imaging console. A 72‐mm‐diameter quadrature volume coil (Rapid Biomedical) was used in transmit/receive mode. A 2‐mm imaging slice was selected through the sample tubes or mice (field of view 80 × 80 mm). Longitudinal (T_1_) relaxation times were measured using an inversion recovery‐FLASH sequence (data matrix 128 × 64, repetition time 5 ms, echo time 3 ms, 26‐s delay between inversions, 9 inversion times between 0.1 and 25.6 s, two acquisitions per inversion time). Transverse (T_2_) relaxation times were measured using a multiecho sequence (data matrix 256 × 128, repetition time 2 s, echo spacing 10 ms, 64 echoes). Imaging data were fitted to exponentials using Matlab software (Mathworks). During imaging mice were kept under anesthesia using 2% Isofluorane in oxygen, and breathing rate was monitored. Body temperature was monitored using a rectal probe, and maintained using heated air.

### Iron Assay

Cellular iron content was assayed using a published protocol 50.

### Electron Microscopy

Cells were grown to ∼80% confluence, washed twice with 0.9% saline, fixed for 2 h on ice in a mixture of 2.5% glutaraldehyde and 2% formaldehyde, and scraped into Eppendorf tubes and pelleted. Fixed cells were rinsed 3 times in 0.1 M PIPES buffer and osmicated by incubating for 1 h in 1% osmium tetroxide with 1.5% potassium ferricyanide. They were rinsed four times in distilled water, bulk stained in 2% uranyl acetate in 0.05 M maleate buffer at pH 5.5, rinsed 3 times in distilled water and dehydrated with graded solutions of ethanol (70%, 95%, and 100%) for 5 min in each solution. They were then incubated in 50% acetonitrile 50% epoxy resin, without a catalyst. This was followed by five daily changes of pure resin with no catalyst. Samples were then placed in coffin molds in degassed resin and air was excluded by covering the resin with a thin Aclar sheet (Agar Scientific) and cured at 60°C for 24 h. Fifty nanometer sections were cut and mounted on bare 300‐mesh copper grids and viewed using a Philips CM100 transmission electron microscope operated at 80 kV.

### Toxicity Assay

To measure the effect of reporter gene expression and uptake of ferritin and manganese‐loaded ferritin on cell growth, the proliferation rate of the cells was measured over the space of a week. Cells expressing the transgene, and untransduced cells, were seeded into wells of a six‐well plate (Nunc), and incubated in either growth media, or growth media containing 75 nM ferritin or manganese‐loaded ferritin. Growth rate was determined by measuring the percentage area of each well covered by cells at 3‐hourly intervals using an Incucyte system (Essen Bioscience). Cells were allowed to grow for 24 h before addition of the contrast agent, to control for cell growth under normal conditions. Cell viability was assessed using Trypan blue dye exclusion using an automated Vi‐cell (Beckman‐Coulter) counter, according to the manufacturer's instructions.

### Animal Studies

Female mice with severe combined immunodeficiency (SCID) were purchased from Charles River, UK. At the time of cell implantation, animals were aged between 6 and 8 weeks and weighed between 20 and 22 g. Xenografts, which were obtained by implanting 1 × 10^7^ HEK 293T cells suspended in 100 µL PBS in each flank, were imaged at 14 days after cell implantation. Procedures were carried out under the authority of project and personal licenses issued by the Home Office, UK, and were approved by local Animal Welfare and Ethical Review Bodies.

## RESULTS

### Timd2 Expression Mediates Uptake of Ferritin

**Figure 1:**
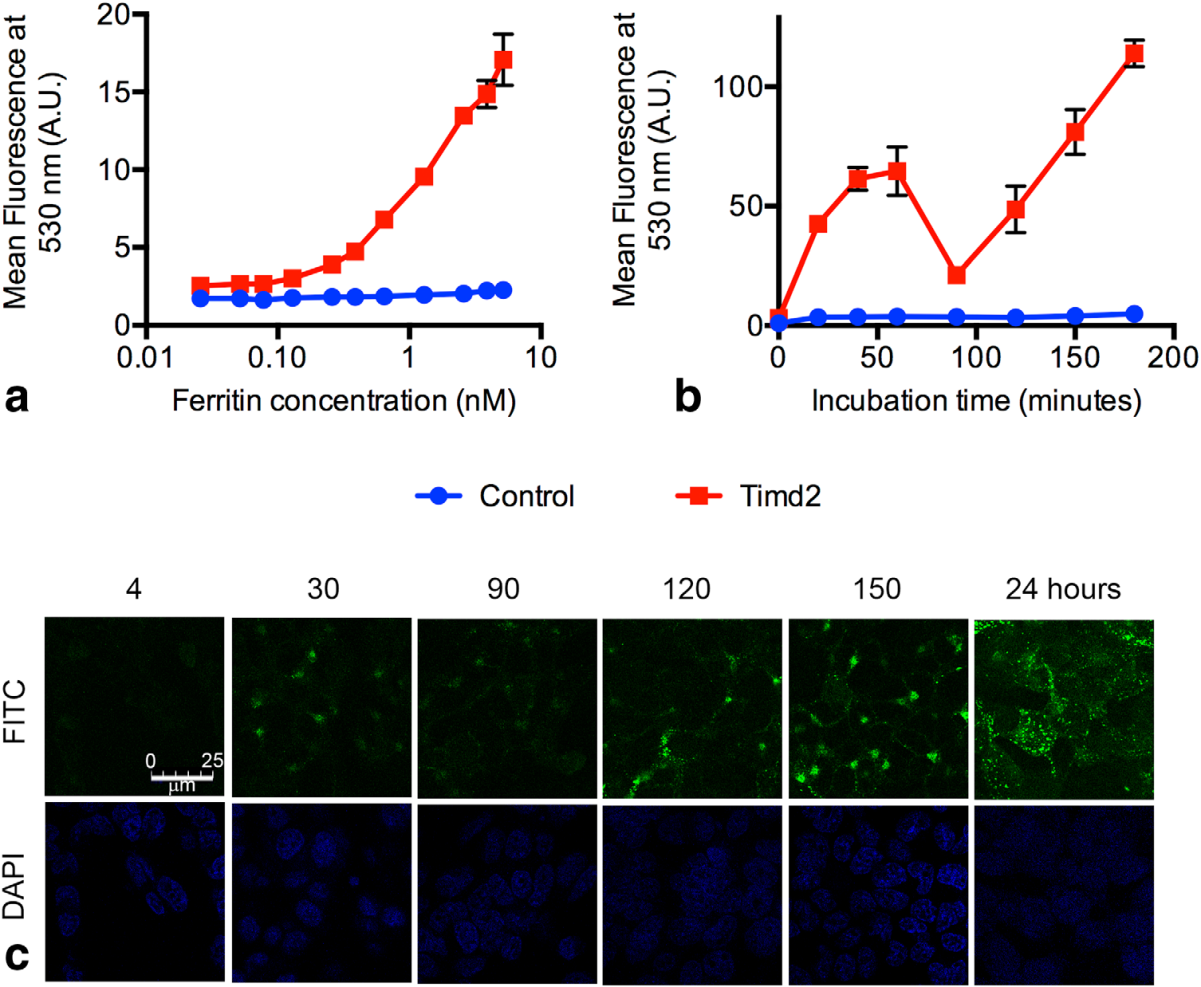
Uptake of labeled ferritin by Timd2‐expressing cells. **a**: Fluorescence of Timd2‐expressing cells and control cells after 3 hours of incubation with the indicated concentrations of FITC‐labeled ferritin. **b**: Fluorescence of Timd2‐expressing cells and control cells incubated for the indicated times with FITC‐labeled ferritin at a concentration of 75 nM. Fluorescence of the cells in (a) and (b) was measured using flow cytometry, where the points represent the mean fluorescence of four independent populations of 10000 cells, with the mean for each population representing the mean fluorescence of the total cell population. Error bars show the standard deviation of the means for the different cell populations. Some error bars are obscured by the data points. **c**: Confocal microscopy images showing Timd2‐expressing cells following incubation with FITC‐labeled ferritin for the indicated times in minutes and at 24 h.

Previous studies have shown that ferritin is endocytosed by Timd2‐expressing cells and processed by means of the endocytic pathway into an acidic perinuclear compartment within 30 min 40, 41, 51, 52. To confirm this mechanism of uptake HEK 293T cells, which had been transduced with a lentiviral vector expressing mStrawberry and Timd2, were incubated with FITC‐labeled ferritin and ferritin uptake followed using flow cytometry and confocal microscopy (Fig. 1). There were concentration‐ (Fig. 1a) and time‐dependent (Fig. 1b) increases in mean fluorescence intensity following incubation of cells with FITC‐labeled ferritin but no changes in fluorescence in control cells not expressing the receptor.

The intensity of fluorescein fluorescence is strongly pH dependent 53, 54 and has been used to study protein trafficking through organelles 55 and the increasingly acidic endocytic pathway 56, 57, 58, 59. In cells incubated with a fixed concentration of FITC‐ferritin (75 nM) there was an increase in fluorescence between 0 and 40 min of incubation, which then remained relatively constant for the next 20 min, before decreasing over the next 30 min and then steadily increasing again thereafter (Fig. 1b). FITC is most fluorescent above pH 7, decreasing to below 40% of its maximum level at pH 6, 20% at pH 5.5, and less than 10% at pH 4 54. Previous investigations have described the endocytosis of ferritin as it goes through the endocytic pathway: first to the early endosome (pH ∼6) 51, which it can reach in as little as 2 min 40, then the late endosome (pH 5‐6), and finally to the lysosome (pH 5.5‐4) 57, 60, where the iron core is broken down 61, 62, 63. Together with these previous studies, the results presented in Figure 1b suggest that ferritin accumulates in an acidic compartment, which causes a decrease in fluorescence after 60 min. The subsequent increase in fluorescence is likely to be due to recycling of Timd2 receptors to the plasma membrane, initiating further uptake, and/or release of FITC‐labeled peptides from the acidic lysosome 60. Confocal microscopy (Fig. 1c) showed that after 4 min ferritin appeared bound to the plasma membrane and also in several small endosome‐like compartments, consistent with previous reports of ferritin uptake to endosomes within 2 min of incubation 60, 64. After 30 min, ferritin was seen only in punctate cytoplasmic structures, suggesting that all the available Timd2 receptors on the plasma membrane had been endocytosed. FITC fluorescence then decreased at 90 min, and appeared to be located around the nucleus, consistent with a peroxisomal location and the results shown in Figure 1b. At later times the fluorescence increased (Fig. 1c), as was observed with the flow cytometric studies, suggesting that the Timd2 receptors had been recycled and that further ferritin endocytosis had occurred.

### Timd2 Expression Is Detectable Using T_2_‐Weighted Imaging

**Figure 2:**
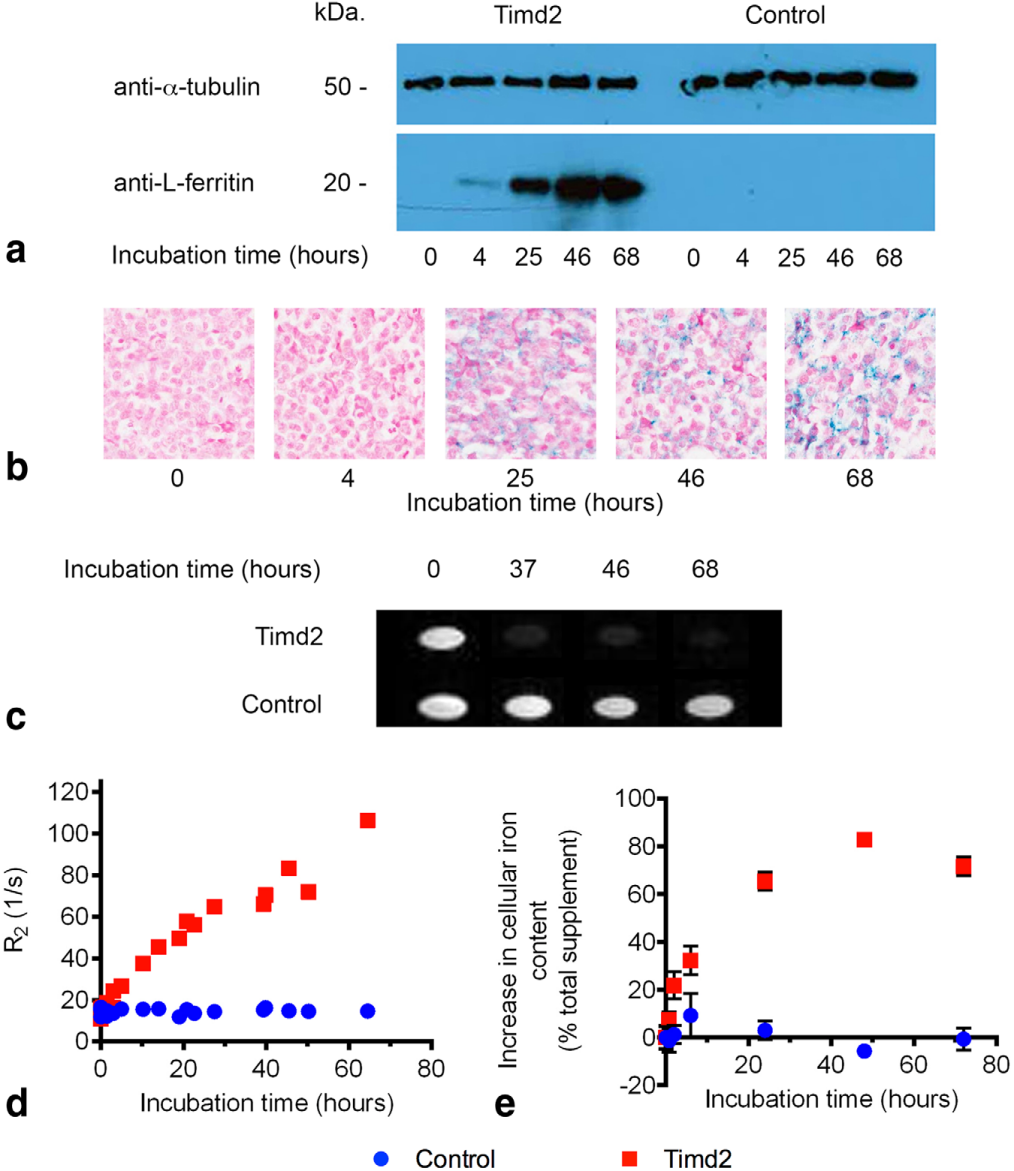
a: Western blot showing increase in ferritin light‐chain content of Timd2‐expressing cells, but not in untransfected control cells, following incubation with ferritin (75 nM). Bands indicating the presence of ferritin in untransfected control cells were detectable after longer exposure times (not shown). **b**: Perls' Prussian blue staining of fixed Timd2‐expressing cells showing increasing iron content with length of incubation with ferritin (75 nM). **c**: T_2_‐weighted images of pellets of Timd2‐expressing and control cells that had been incubated for the indicated times with 75 nM ferritin (TR = 2000 ms; TE = 20 ms; slice thickness = 2 mm). **d**: R_2_ of Timd2‐expressing and untransfected control cells incubated for the indicated times with 75 nM ferritin. R^2^ = 0.9465 for Timd2‐expressing cells and R^2^ = 0.026 for control cells. The baseline R_2_s before ferritin incubation were 13.45 ± 2.13 and 14.9 ± 2.09 in Timd2‐expressing and untransfected control cells respectively (n = 4; ±SD). **e**: Iron content in Timd2‐expressing cells and untransfected control cells after incubation for the indicated times with 75 nM ferritin. Cellular iron content is displayed as a percentage of the total iron content of the added ferritin. Points represent the mean of 3 independent replicates, error bars show ±SD. The baseline iron content in untransfected control cells and Timd2‐expressing cells was 0.51± 0.05 and 0.42 ± 0.05 pg Fe/cell, respectively. This rose to 0.61 ± 0.1 and 1.31 ± 0.03 pg Fe/cell at their respective peaks at 6 and 48 hours of incubation with ferritin.

Incubating cells with 75 nM ferritin resulted in an increase in the levels of intracellular ferritin, as determined by western blotting (Fig. 2a) and a corresponding increase in iron content, as determined by staining cells with Perl's Prussian blue (Fig. 2b), in Timd2‐expressing cells but not in control cells. Cell pellets imaged at 7T showed an increase in R_2_ in Timd2‐expressing cells but not in control cells (Fig. 2c,d). There was a linear increase in R_2_ for Timd2‐expressing cells with time, over a period of nearly 70 h (R^2^ = 0.9465), while there was no significant increase in control cells (*P* = 0.5; r^2^ = 0.026) (Fig. 2d). The relaxation enhancement in Timd2‐expressing cells peaked with a >seven‐fold increase in R_2_ above baseline. The increase in R_2_ in Timd2‐expressing cells was associated with an increase in iron (Fig. 2e) and ferritin contents (Fig. 2a). These data are consistent with previous findings that R_2_ increases linearly with iron‐bound‐ferritin concentration in solution 65.

**Figure 3:**
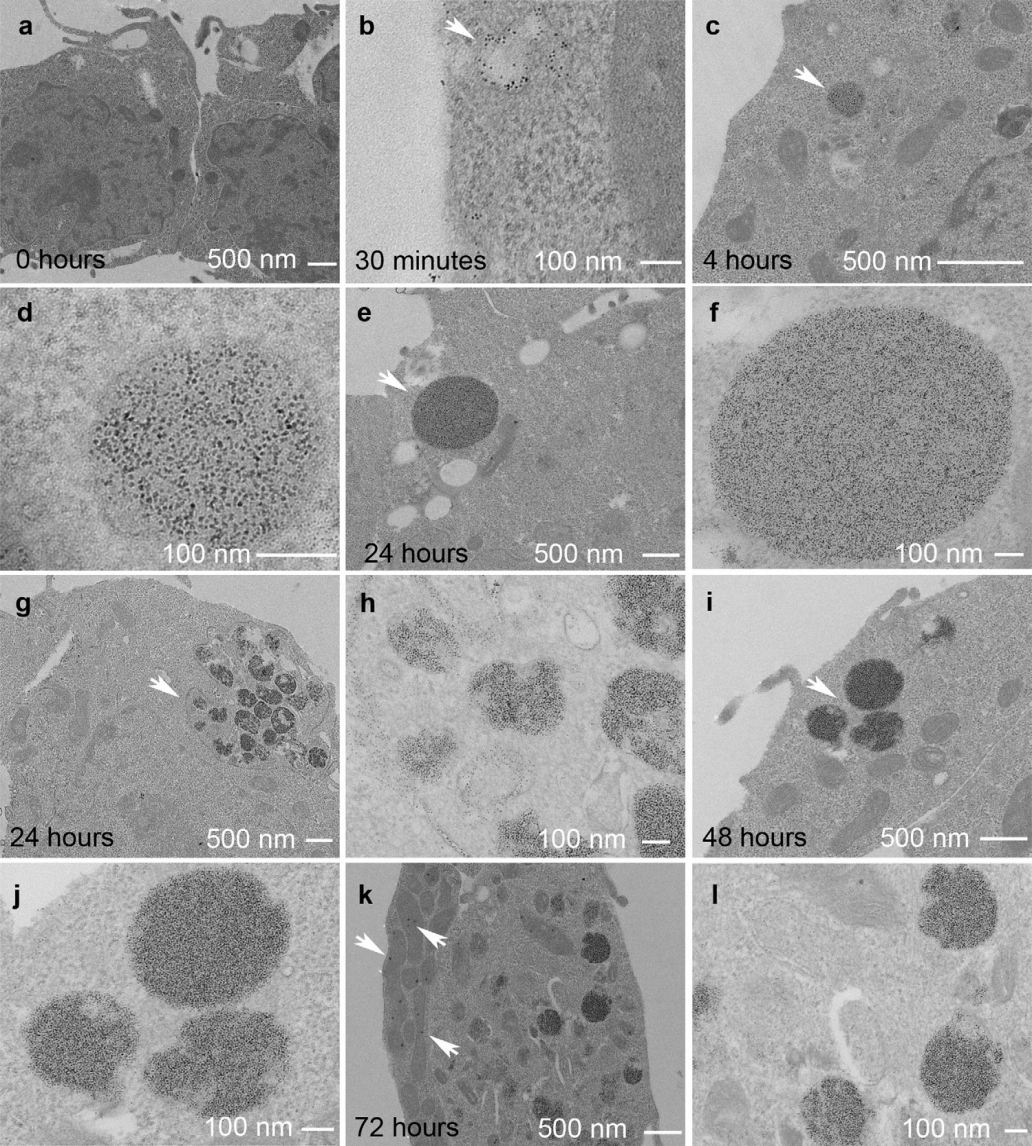
Representative transmission electron micrographs of Timd2‐expressing cells. **a**: No incubation with ferritin. **b**: Following 30 min incubation with 75 nM ferritin, arrow indicates endocytic vesicle containing ferritin. **c**: Four‐hour incubation, arrow points to a lysosome containing ferritin. **d**: Four‐hour incubation. Image of arrowed structure in c at higher magnification. **e**: Twenty‐four‐hour incubation, arrow shows large lysosome containing ferritin. **f**: Image of arrowed structure in (e) at higher magnification. **g**: Twenty‐four‐hour incubation, arrow points to a group of multivesicular bodies and lysosomes, surrounded by tubular endosome. **h**: Higher magnification image of the arrowed structure in g. Ferritin can be seen in the cytoplasm, multivesicular bodies, lysosome, and tubular endosome. **i**: Forty‐eight‐hour incubation, arrow points to a group of three ferritin‐containing lysosomes. **j**: Image of arrowed structure in (i) at higher magnification. Ferritin is more densely packed within the lysosome than at 24 h. **k**: Seventy‐two‐hour incubation. Electron dense accumulations are also prominent in the mitochondria, see arrows. **l**: Higher magnification image of (k), showing three lysosomes containing ferritin. Ferritin is also visible in the cytoplasm.

Transmission electron micrographs of Timd2‐expressing cells that had been incubated under the same conditions as used for the MRI experiments, showed no aggregation of ferritin initially (Fig. 3a), but after 4 h ferritin aggregates of up to 200 nm in diameter could be seen (Figs. 3c,d). By 24 h aggregates over 0.5 μm in diameter had formed (Figs. 3e–h), which appeared denser at 48 and 72 h. Ferritin aggregates were also visible in cells stained with Perls' Prussian blue (Fig. 2b).

### Timd2‐Mediated Mn‐Loaded Apoferritin Uptake Enhances T_1_ and T_2_ Relaxation

**Figure 4:**
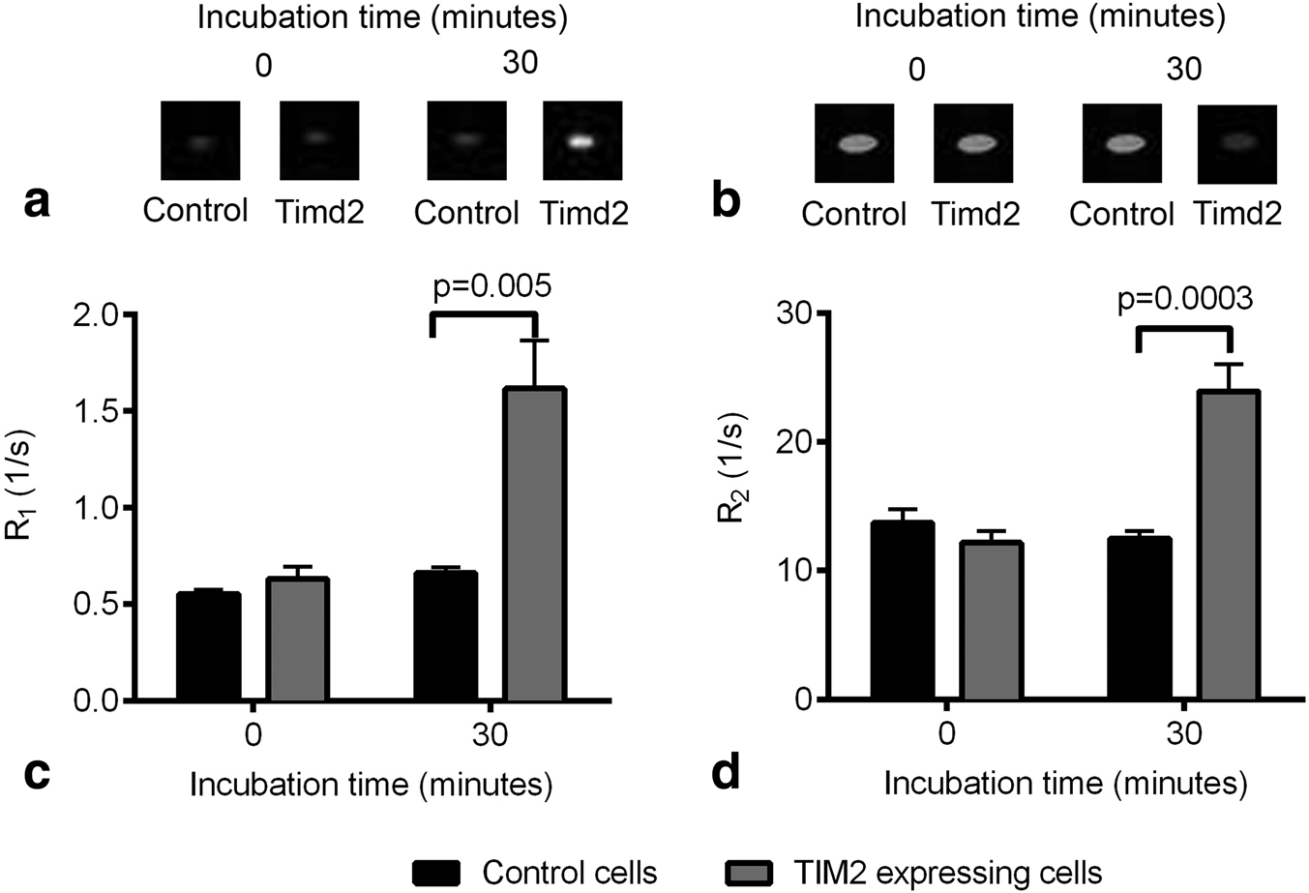
a: T_1_‐weighted images of pellets of Timd2‐expressing and control cells that had been incubated with 75 nM Mn‐apoferritin for 30 min. (TR = 5 ms TE = 3 ms). **b**: T_2_‐weighted images of cell pellets of Timd2‐expressing and control cells that had been incubated with 75 nM Mn‐apoferritin for 30 min. (TR = 2 s TE = 30 ms). **c**: Changes in R_1_ in Timd2‐expressing and control cells after 30 min of incubation with 75 nM Mn‐apoferritin. Points show the mean of at least 4 results, error bars show ±SEM. **d**: Changes in R_2_ in Timd2‐expressing and control cells after 30 min of incubation with 75 nM Mn‐apoferritin. Points show the mean of at least 4 results, error bars show ±SEM.

Manganese is an effective T_1_ contrast agent due to the paramagnetism of its five unpaired electrons and can form solid MnOOH mineral cores in apoferritin 42. Images of cell pellets obtained from cells that had been incubated for 30 min with Mn‐apoferritin showed that signal was increased in T_1_‐weighted images (Fig. 4a) and decreased in T_2_‐weighted images (Fig. 4b) in cells expressing Tim2d but not in control cells. This corresponded to a significant increase in R_1_ (*P* = 0.005) (Fig. 4c) and R_2_ (*P* = 0.0003) (Fig. 4d) in cells expressing Timd2, while there was no change in control cells.

### Effect of Timd2 Expression and Incubation with Ferritin on Cell Growth and Viability

**Figure 5:**
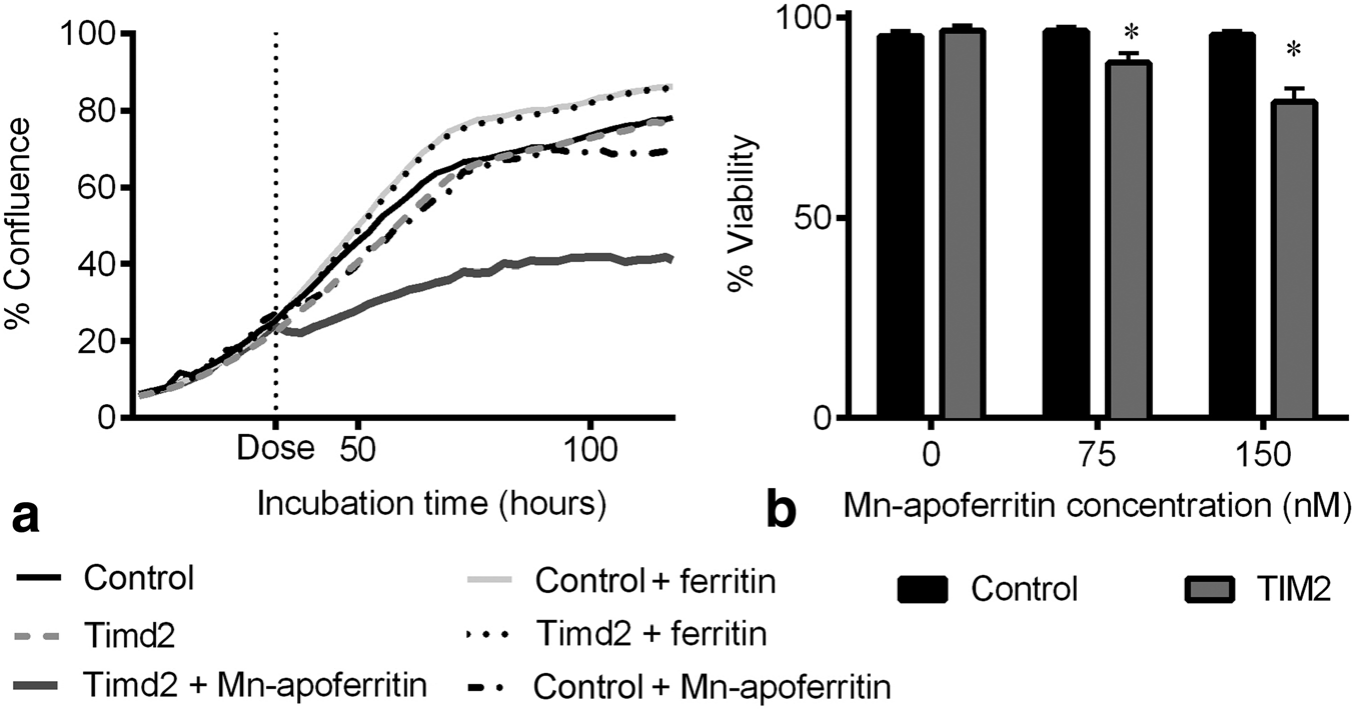
Effect of Timd2 expression and incubation with ferritin or manganese‐loaded ferritin on cell growth. Cell growth is expressed as the percentage cell coverage of the well. **a**: After 30 h of cell growth Timd2‐expressing and control cells were incubated with 75 nM Mn‐apoferritin, or ferritin, as indicated by the vertical dotted line. **b**: Effect of Timd2 expression and incubation with ferritin or manganese‐loaded ferritin on cell viability.

Expression of Timd2 had no effect on the growth of HEK 293T cells when compared with nontransduced control cells (Fig. 5a) and incubation of these cells with 75 nM ferritin produced a slight increase in their rate of proliferation. In contrast, incubation of Timd2‐expressing cells with 75 nM Mn‐apoferritin markedly inhibited their rate of proliferation and decreased their viability (Fig. 5b), whereas it had no significant effect on the growth or viability of control cells.

### Detection of Timd2‐Expressing Cells In Vivo Using T_2_‐Weighted Imaging

**Figure 6:**
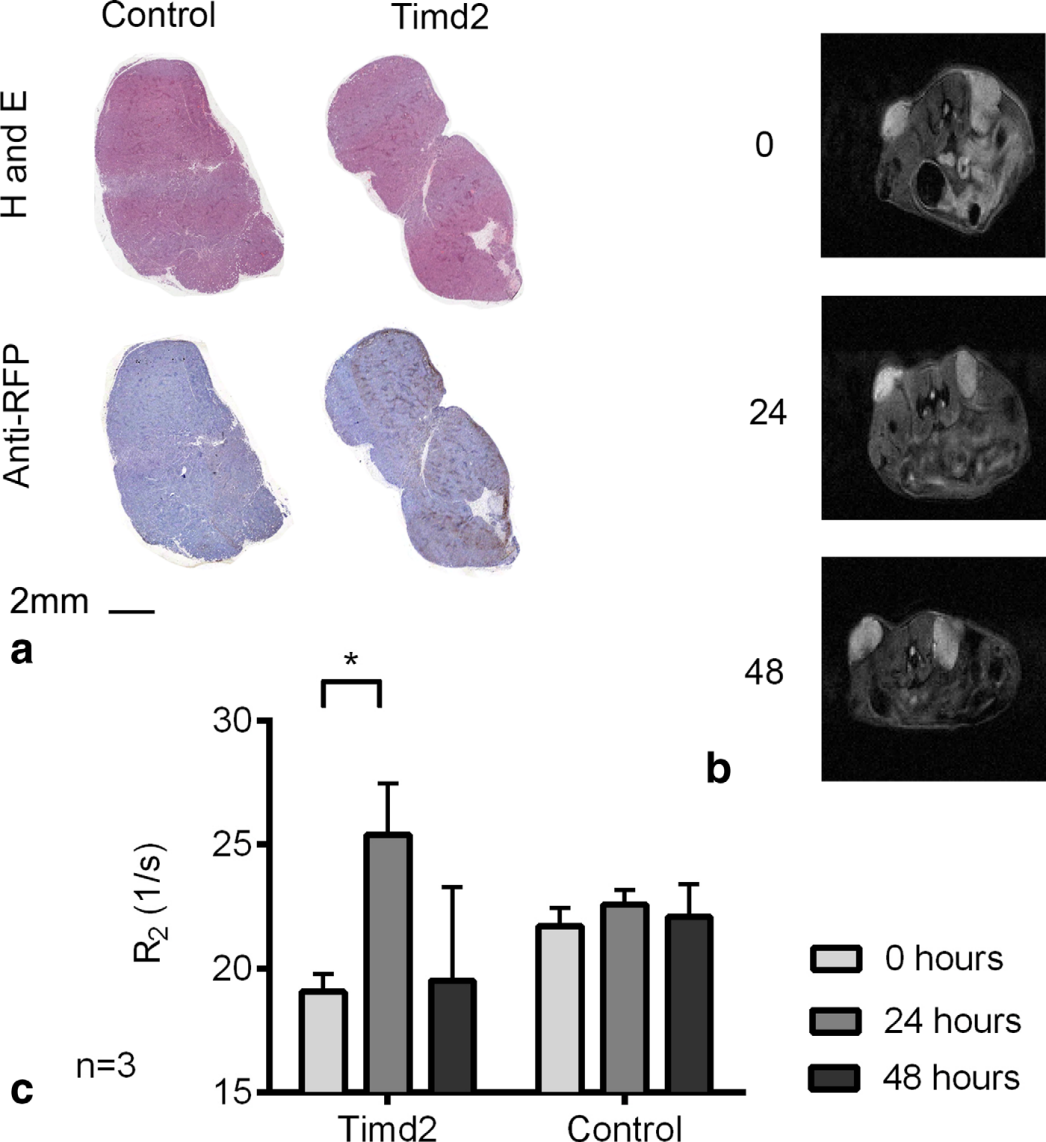
Effect of ferritin injection on T_2_ in Timd2‐expressing cells in vivo. **a**: Representative H&E stained tissue sections from control and Timd2 xenografts obtained 14 days after cell implantation. Timd2 expression was confirmed by staining with an anti‐RFP antibody. **b**: T_2_‐weighted images from a representative mouse from (a), showing control xenograft (left) and Timd2‐expressing xenograft (right) before, 24 and 48 h after ferritin injection. **c**: Changes in T_2_ in Timd2‐expressing tissue and controls at the indicated times after injection of ferritin. The change in R_2_ was significant in Timd2‐expressing tissue but not in control tissue 24 h after ferritin injection *P < 0.05 (1‐tailed paired t‐Test, n = 3). R_2_ values before ferritin injection for Timd2‐expressing and control xenografts were 19.01 ± 1.27 and 21.7 ± 1.28, respectively.

Xenografts were produced by implanting untransfected control cells and mStrawberry‐Timd2‐expressing HEK 293T cells subcutaneously in the contralateral flanks of mice and imaged at 14 d after implantation. Expression of Timd2 was confirmed by staining tissue sections obtained post mortem with an anti‐red fluorescent protein (RFP) antibody that recognizes mStrawberry (Fig. 6a). The coding sequences for mStrawberry and Timd2 in the viral vector were separated by an E2A sequence, which ensures that they are coexpressed 45. Hematoxylin and eosin (H&E) staining showed that the tissue architecture and levels of necrosis were similar in the control xenografts and in the xenografts expressing Timd2, consistent with Timd2 expression having no measurable effect on the growth and viability of these cells in vitro (Fig. 5). The xenografts were imaged before i.v. administration of 11.2 mg ferritin and then again at 24 and 48 h, using a T_2_‐weighted imaging sequence. Representative images are shown in Figure 6b. There was a significant increase in R_2_ in Timd2‐expressing tissue after 24 h, although this had disappeared by 48 h.

## DISCUSSION

We have shown that Timd2‐expression facilitates the cellular accumulation of ferritin and manganese‐loaded apoferritin, leading to large enhancements in R_2_ and R_1_ in vitro. The peak R_2_ enhancement observed here of greater than seven‐fold at 7T is better than or comparable with enhancements measured previously in vitro in cells expressing other T_2_‐based reporter genes, including a four‐fold increase in R_2_ at 3T in 293FT cell pellets expressing the MagA transgene 6, a two‐ to three‐fold increase at 7T in cells coexpressing the ferritin and transferrin genes 4, and a two‐fold increase in R_2_, produced by a chimeric re‐engineered ferritin reporter with improved iron loading, after 24 h of incubation with iron 66. The increase in R_2_ observed here was much greater than that expected from the amount of ferritin added to the cells. Assuming an R_2_ of 1500 s^−1^mM^−1^ for ferritin, which was measured at 7T in HEPES buffered saline, and that all the added ferritin was present in the cell pellet, then the expected increase in R_2_ is 26 s^−1^ above a background R_2_ of around 14 s^−1^, which means that the peak R_2_ should be ∼40 s^−1^. This is much lower than the maximum R_2_ observed in Timd2‐expressing cells of 116 s^−1^, which represents an increase in R_2_ of over 100 s^−1^, and four‐fold greater than the predicted increase. A possible explanation for this unexpectedly large increase is the ferritin aggregation that we observed in the electron micrographs (Fig. 3), which can increase R_2_ for ferritin by up to five‐fold 67.

An unexpected observation was that the baseline R_2_ in Timd2‐expressing cells was lower than in control cells, both in vitro and in vivo (Figs. 2d,6c), although the differences were not significant. Consistent with a lower R_2_, Timd2‐expressing cells also had 18% less iron than control cells before the addition of ferritin, although again the difference was not significant (Fig. 2e). The reasons for this are unclear.

Incubation of Timd2‐expressing cells with 75 nM manganese‐loaded apoferritin resulted in a nearly three‐fold increase in R_1_ after only 30 min of incubation and a near doubling of R_2_, with no significant change of R_2_ in the controls (Figs. 4a,c). The three‐fold increase in R_1_ seen here in Timd2‐expressing cells is similar to that observed in hepatocytes incubated with manganese or with manganese‐loaded transferrin 68, where R_1_ was increased to 1.6 s^−1^ after 2 h incubation with 31.5 µM Mn(III)‐transferrin. The changes in R_2_ observed following prolonged incubation with iron‐loaded ferritin (Fig. 2) suggest that further increases in R_1_ would be obtained by extending the incubation time with manganese‐loaded apoferritin. However, these experiments were not pursued in view of the evident toxicity of this contrast agent, which was only observed in Timd2‐expressing cells and not with iron‐loaded ferritin (Fig. 5). There are few reports of other T_1_‐based reporter genes with which our results can be compared directly; however, in a recent study using another reporter, which used the same cell type and promoter, we showed increases in R_1_ of three‐ to seven‐fold in vivo 16. This reporter system based on Oatp1 and gadoxetate may be preferable to manganese‐based systems not only because of the higher levels of contrast that have been achieved but also because of the lower toxicity of this clinically‐approved contrast agent.

Xenografts produced by subcutaneous injection of Timd2‐expressing cells also showed significant increases in R_2_ in vivo following i.v. injection of ferritin, although the increases were much lower than those observed in vitro (Fig. 6). This could be due to several reasons, including rapid clearance of ferritin from the blood (the blood half‐life in rats has been reported at being between 4 and 30 min) 69, 70 and poor delivery of ferritin to the tissue. With respect to the latter point, reporters that rely on the use of small molecule contrast agents may have an advantage 16, 27. Contrast may be improved by changing the timing of imaging after ferritin injection. Although ferritin is cleared rapidly from the circulation, we delayed imaging until 24 h to give sufficient time for ferritin trapped nonspecifically by the enhanced permeability and retention effect 71 to clear from the tissue. However, imaging at earlier times after injection may give better contrast. Contrast may also be improved by giving multiple ferritin injections, because it is clear that Timd2‐expressing cells will continue to accumulate ferritin over prolonged periods of time (Fig. 2). Consistent with the literature, our data suggest that ferritin is trafficked to acidic endosomal and lysosomal compartments after uptake (Figs. 1b,c), where it has been shown to demineralize and release free iron 61, 62, 63. As endogenous ferritin expression is controlled at both the transcriptional and translational level by the concentration of free iron in the cell, this would be expected to lead to the synthesis of new ferritin 72. This process could explain the lag we observed between peak iron content and the peak increase in R_2_ in vitro (Figs. 2d,e). Coexpression of Timd2 with a ferritin transgene may therefore produce even greater changes in contrast than those observed here, where we have relied on endogenous iron homeostasis mechanisms to repackage the degraded ferritin 4. Although manganese‐loaded apoferritin would also be expected to demineralize during uptake [manganite breakdown occurs between pH 6 and pH 4 73, while ferrihydrite breakdown in ferritin occurs between pH 5 and pH 2 74], incorporation of manganese into native ferritin would not be expected as this does not occur below pH 8.5 42. A recent study investigated coexpression of the manganese‐binding protein MntR together with the manganese importer DMT1, which has previously been shown to function as a gene reporter 15. However, coexpression did not lead to significant further contrast enhancement in vivo despite promising results in vitro 75. Expression of MntR may also provide a means of reducing the toxicity of free manganese by binding it, however, its effect on reducing toxicity was not investigated 75. Recent work suggests that changes in T_1ρ_ may be a more sensitive indicator of the presence of superparamagnetic material in tissue 76, although this has yet to be demonstrated in vivo.
